# Learnings from conducting mental health research during 2004 tsunami in Tamil Nadu, India

**DOI:** 10.1186/s12889-020-09733-y

**Published:** 2020-10-29

**Authors:** R. Padmavati, Vijaya Raghavan, Heather Rera, Megan Kearns, Kotteeswara Rao, Sujit John, R. Thara

**Affiliations:** Schizophrenia Research Foundation, R/7A, North Main Road, Anna Nagar West Extension, Chennai, Tamil Nadu 600101 India

**Keywords:** Research, Tsunami, Mental illness, Tsunami, Disaster management, Disaster psychology, Crisis response, Evaluation, LMICs

## Abstract

**Background:**

The Indian Ocean tsunami experience in 2004 caused a major loss of life and subsequent emotional trauma for survivors. Psychosocial needs in the aftermath of this disaster were extensive, yet the cohesion and effectiveness of response were limited due to lack of preparedness and relevant policy. The Schizophrenia Research Foundation (SCARF) was one of the organizations that quickly responded to provide psychosocial assistance to people in the tsunami affected areas and recognized a need for relevant research on disaster response. Therefore, we undertook research on the challenges, success and limitations of psychosocial interventions in alleviating post-traumatic symptomology.

**Discussion:**

Both community-level workers and researchers were limited in their preparedness to carry out tasks related to response. Language barriers, cultural differences, and a gap in long-term services limited the breadth and scope of research that was able to be completed. Lack of policy, poor co-ordination of services, lack of trained researchers and limited resources were challenges that emerged during this period and various strategies were adopted to meet these challenges.

**Conclusions:**

Continued research and evaluation of data has brought crucial considerations to light, including the variance in symptomology, effective tools of measurement, and the nuanced response of survivors. Future research should take relevant factors into consideration including barriers to care. Understanding of the local language and religious beliefs are significant resources in understanding the nature of survivors’ trauma response and effective means of coping. Lastly, limitations regarding time frame and scope of research should be evaluated to provide more effective, comprehensive methods in future studies.

## Background

### Humanitarian context

This research was conducted during the aftermath of the Indian Ocean tsunami on December 26, 2004. An undersea earthquake that struck off the coast of Indonesia caused the ocean floor to suddenly rise, triggering a massive tsunami. A succession of waves proceeded to hit the coastlines of Thailand, India, Sri Lanka and South Africa. The states of Tamil Nadu, Andhra Pradesh, Kerala, and Andamans were most affected by the impact of the tsunami, with the highest loss of life, estimated at 7951 lives lost. In the state of Tamil Nadu [1].

As in any disaster, the needs of citizens in the aftermath of the tsunami were extensive. Aid organizations led with rescue and physical needs as a priority, but resources exploring psychological impacts were sparse. Due to the close proximity of this disaster and the understanding of possible mental health needs, Schizophrenia Research Foundation (SCARF) was one of the organizations that quickly responded to provide psychosocial support and mental health services (PSSMHS) to people in the tsunami affected areas. While providing the services for a period of period of 4 years (2004–2008), SCARF identified an opportunity for research, the findings of which could be critical to future disaster response and preparedness.

### Research study

The main objectives of the study were: a) understand needs of the persons in the affected areas and improve the design and implementation of interventions, informed by the research during disaster, with focus on the population in the tsunami-affected areas with pre-existing mental illnesses and those with emerging mental health issues caused by the disaster; and b) evaluate the effectiveness of training community health workers in psychosocial strategies.

Based on the observations and data collection by the SCARF team during field visits to the affected areas, staff formulated training materials and a psychosocial intervention program which was delivered in the study sites. The strategies implemented by SCARF included: needs assessment, development of manuals in counseling, a short film on tsunami psychosocial interventions, a training program for community level workers (CLWs) and a community mental health program including a community clinic using videoconferencing. CLWs were supported financially though the funds received by SCARF from humanitarian organizations around the world to support and provide psychological support to the affected people.

The outcomes of these interventions were periodically evaluated, alongside the effectiveness of interview strategies for collection of data. Over half the population surveyed indicated the need for some kind of psychosocial support [2]. The researchers found that combining a needs assessment with resources or immediate interventions was the most supportive, and therefore, widely accepted approach, and should be documented in a standardized manner. Evaluating the training programmes for psychosocial interventions indicated several positive recommendations to be incorporated into future programmes in terms of structure, focus on vulnerable groups and training methods.

## Discussion

### Scientific importance of this research

#### Mental health in India

Mental health has historically been neglected, notably in low and middle-income countries (LMIC). India struggles to maintain the workforce needed to provide treatment to individuals with mental illness, even during times of stability, despite neuropsychiatric disorders accounting for 11.6% of the national burden of disease [3, 4]. The mentally ill often need to advocate for themselves, or have others do so, in order to get their needs met. During disaster, it becomes extremely difficult for this workforce to deliver mental health care urgently, resulting in the mental health needs of the population remaining largely unmet. In India, the Disability Adjusted Life Years (DALYs) lost to a mental illness is higher than those of many common physical illnesses [5], justifying greater attention to these diseases. Yet there remains a deficit in funding and practice to meet these needs. In times of crisis, this population goes without necessary support, creating barriers in access to food, medical help, and other basic needs.

#### Research on humanitarian crises and disaster psychology from India

Many research studies on mental health have been undertaken by different organizations during various humanitarian crises and natural disasters that occurred in India [6–12], especially with mental health [13]. One of the most important and extensively studied disaster is the Bhopal gas tragedy in 1984 and the affected population are followed-up till date [14–17]. Mental health impact of the Bhopal gas tragedy on the survivors and the general population have been studied longitudinally [18]. The ongoing COVID-19 pandemic, which has affected the whole world, has posed serious physical, mental and social crises to general population in India, especially to the migrant workers [19, 20].

#### Relevance of disaster research

Research specific to a humanitarian crisis permits the creation of relevant tools to be utilized in times of disaster, both for those with chronic mental illness, and individuals who develop symptoms in the aftermath of trauma. The high risk of natural disasters in the Indian sub-continent suggests the necessity for disaster interventions [6]. Crises will inevitably occur, and it is part of responsible disaster preparedness to create sustainable interventions to be utilized during these times. Research by SCARF, disseminated through practice and two publications, described relevant needs and observations [2, 21]. Several important findings emerged. A rapid needs assessment in the affected areas of Chennai identified counselling and medication as important needs. Evaluation of the training programs indicated significant utilisation of the training, expressed need for structure and reinforce sessions, and the need for a list of mental health services for easy access.

### Challenges to research

Identifying the challenges to research remains crucial to understand approaches to future disasters. The absence of relevant and accurate data limits possible interventions for this vulnerable population. Research in the psychosocial component of disaster management is neglected.

#### Lack of policy for mental health research as a part of disaster interventions

The workers involved in disaster recovery are often proficient in assessing physical health needs and available resources but have limited understanding of mental health and illness. There were no policy documents that aided research in psychosocial interventions during disaster situations.

#### Poor coordination

In the aftermath of tsunami, there was an excess of volunteers but a lack of coordination. SCARF’s experience was that a research-without-intervention approach frustrated survivors and instilled a resistance to participation in future research. NGOs worked independently of each other and resources and data kept within each organization. As survivors were moved from shelter to shelter, their information failed to move with them, forcing staff to collect data again. This lack of corroboration between NGOs led to understandable frustration in survivors.

#### Limited researcher training and research capacity

Disasters demand a considerable workforce to provide services, with thousands from various regions traveling to provide aid. Lack of awareness of cultural norms and proficiency in the local language, were inadvertent barriers to service delivery. This further led to a lack of a unified method of data collection and limited the scope and utilization of the completed research. It is necessary for researchers to be proficient in relevant topics related to disaster recovery. Lack of clarity on what to measure led to elongated questioning on irrelevant topics, a wasteful exercise given the lack of human resources to conduct research [2]. If goals were synthesized and researchers trained in specific measures, research would be more efficient and relevant to disaster recovery.

#### Limited resources for referral

In SCARF’s experience, when assessment indicated the need for further intervention, professionals struggled to find resources for referral due to the lack of governmental facilities and coordinated care, presenting a large ethical issue [21]. Rapidly changing location of recovery sites, frequent movement of participants, and restricted physical boundaries approved for research limited collection of subsequent data. Individuals with history of mental illness, and those with current issues, illustrated a need for further care and medication to manage his or her symptoms. Without a place to refer these individuals, the cycle of evaluation without intervention perpetuates. This limits future research that could be done to follow-up with patients, review records of treatment and their experience in an affiliated hospital, which could provide longitudinal data.

### Strategies used to address the challenges

#### Adapting community mental health interventions

Based on several decades of work in the community, existing strategies were redesigned to tackle various unforeseen scenarios following the tsunami. These included treating patients with chronic mental illness, individuals who discontinued mental health treatment, and persons with new-onset psychological symptoms. Local temples, mosques, and churches were places that people accessed to find healing and peace. Adding mental health resources in those settings reduced stigma surrounding care [21]. Operating mental health services in traditional healing sites also helped to increase mental health services [22]. The re-designed strategies were evaluated for relevant outcomes. Researchers in the field were provided training in data collection aimed at evaluation.

#### Training community level workers (CLWs)

SCARF worked with governmental and non-governmental organizations to increase recovery options. This included training CLWs on psychosocial intervention methods that did not require a background in psychology. Studies have shown that it is cost effective to introduce task-shifting, thus reducing burden on the limited number of existing care facilities. When supervised by mental health specialists, non-specialist health professionals and lay workers are able to “detect, diagnose, treat, and monitor individuals with mental disorders and reduce caregiver burden” [23, 24]. Training CLWs also ensures that for long-term disaster management, when incoming aid inevitably leaves, trained workers remain within the community to provide care and support [21].

Care offered to survivors was often one-on-one interactions, while some received medications from nearby hospitals, and group activities. CLWs, who were from the local areas, were discouraged from talking about the tsunami and encouraged to focus attention on daily living and reestablishing lives [25]. The group activities ranged from dance and music programs to role-plays, which worked with children in particular. For people who did not benefit from these interventions, CLWs would walk with them to the beach or temples.

Training in proper documentation was incorporated to enable collection of relevant data to evaluate intervention strategies implemented in the affected areas. Qualitative information was also obtained through individual interviews. With the CLWs. CLWs reported that interventions worked best with children, and that adults were reserved when prompted about their experience.

#### Training volunteer government employees, NGO workers, and university students

The training was related to disaster response psychosocial interventions and disaster management as a whole, based on clear guidelines. Based on the data on the effectiveness of training, recommendations have been made to policy makers for a more collaborative approach, partnerships to share resources and information to aid care and research efforts after a disaster. This approach would increase capacity and efficiency in individual disaster response workers, minimizing the collective burden of care.

#### Telepsychiatry consultations

This enabled addressing the widespread need for services. These consultations bridged the gap for individuals who could not access psychiatric care due to distance from facilities. Consultations took place over a video call, and then medication was dispensed free of cost to the patient with external funding support. Psychoeducation was provided to caregivers and communities aiming to reduce stigma. The data on the clinical care provided was captured in an electronic medical record system and analyzed further [26].

### Informing on policy

Five years after the tsunami, the results of the research done by SCARF were taken note of by policy planners and has begun to inform practice and policy. The National Disaster Management Authority (NDMA) of India now includes psychosocial goals in their disaster plan, specifying implementation of psychosocial support and mental health services [25]. Additionally, Tamil Nadu’s state management plan expanded these suggestions to include specific training of community health workers (CHWs) and other possible interventions in their subsequent revisions, citing work done by SCARF [27]. Lastly, World Health Organization has cited the need for more urgent solutions to societal stigma regarding mental illness, a major barrier to care during stable times and exacerbated in disaster recovery [28].

However, specifics related to research and training are yet to be integrated into practice [22] and address barriers to the commencement of research, such as standardizing ethical guidelines for disaster research and expediting approval from ethical review boards.

### Future direction

Preparedness for mental health services to deal with disaster situations needs to be an integral part of training of those involved in mental health services delivery during crisis. It is imperative that the experience of the past several decades is integrated to develop context-specific guidelines to managing mental health problems during crisis as part of capacity building for crisis. Strategies like technology-based consultations, psychosocial support systems, ease of access to psychotropic medication for persons with serious mental illness and facilitation for emergency care that have been developed [29] needs to be tested and adapted to improve guidelines.

## Conclusion

There were unique challenges that arose specific to the 2004 tsunami regarding how to provide care and collect data. There is a need to develop context-specific strategies to overcome these challenges to maximize healing for those affected after a natural disaster. These strategies will also influence how mental health workers can support themselves, and how to better prepare for future crisis interventions.

This research was conducted during one of the most severe tsunami events in recent history through thoughtful collaboration between two NGOs. In the future, more collaboration between a variety of NGOs, using researchers trained to collect sensitive data in an ethical way while in camps to navigate issues with consent for unaccompanied minors or persons with disability, will elicit a variety of data, and understanding of what challenges responders and survivors are facing pre, during, and post disaster. Despite limited data, conclusions were made regarding how to improve psychosocial aid in the future, including educational materials for aid workers and the public, reaching out to communities with higher rates of mental illness, preparation for region-specific cultural consideration, as well as dissemination of information for staff on how to deal with vicarious trauma.

Future research should continue to analyze the possible design and implementation of humanitarian interventions during a crisis, and potential challenges that could arise. Research should also be conducted to understand the experience of persons with mental health disorders who are marginalized in the community, and what interventions may mitigate increased limitation to services post-disaster. Additionally, governmental agencies should synthesize disaster plans to include relevant NGOs and include specific information regarding the responsibilities of each organization to avoid future overlap. Specific resources should be allocated to conduct research, so crucial information can be collected without shrinking resources needed for PSSMHS. Funding resources must acknowledge the challenges faced in conducting research crisis situations and invest in on-going health research capacity building, multi-sector partnerships and promote local partners leadership [30], ensuring that work is relevant, efficient, and aligned with larger structural goals. Should these groups be deployed for disaster response, training would have increased their capacity to intervene as well as their confidence in working with the affected population.

## Data Availability

Data sharing is not applicable to this article as no datasets were generated or analyzed during the current study.

## Abbreviations

CHW: Community health worker
CLW: Community-level worker
DALY: Disability Adjusted Life Years
NDMA: National Disaster Management Authority
NGO: Non-governmental organization
LMIC: Low and middle-income countries
PFA: Psychological First Aid
PSS: Psycho-social Support
PSSMHS: Psycho-social support and mental health services
SCARF: Schizophrenia Research Foundation
WHO: World Health Organization
